# Still Hurting: Low Back Pain Presentations in the Emergency Department: A Retrospective Observational Study With a Cross‐Sectional Follow‐Up Survey

**DOI:** 10.1155/prm/8903147

**Published:** 2026-06-19

**Authors:** James Hendrie, Jahar Bhowmik, Hafiz Salih, Daryl Yeak, Abdi D. Osman, George Braitberg

**Affiliations:** ^1^ Emergency Department, Austin Health, Heidelberg, Melbourne, Victoria, Australia, austin.org.au; ^2^ Department of Critical Care, University of Melbourne, Parkville, Melbourne, Victoria, Australia, unimelb.edu.au; ^3^ School of Health Sciences, Swinburne University of Technology, Hawthorne, Melbourne, Victoria, Australia, swinburne.edu.au; ^4^ College of Sports, Health and Engineering, Victoria University, St Albans, Melbourne, Victoria, Australia, vu.edu.au

**Keywords:** analgesia, disability, emergency department, low back pain, opioids, Oswestry Scale

## Abstract

**Background:**

Nontraumatic low back pain is highly prevalent in Australia, affecting 79.2% of adults and accounting for up to 4% of emergency department (ED) presentations. This study examines the postdischarge outcomes of ED patients admitted to a short stay unit (SSU) for low back pain management.

**Method:**

A cross‐sectional observational study design was employed, comprising retrospective observational data and a follow‐up cross‐sectional survey. Data were collected between June 2023 and February 2024. Functional status was measured with the Modified Oswestry Low Back Pain Disability Questionnaire.

**Results:**

Of the 422 participants invited, 21% (*n* = 89) completed questionnaires. Respondents were 58% female and 42% male, aged 20.3–96.1 years (mean 63.4, SD 19.4). Most respondents (71.2%) reported moderate‐to‐severe disability, with 19.1% reporting complete disability. Over 50% were still taking opioid analgesics at the time of survey completion. In the multivariable regression model, pharmacological treatments were significantly associated with disability scores (*F* (4, 84) = 5.34, *p* < 0.001). Use of short‐acting opioids was associated with an average increase of 7.5 units in disability score and use of long‐acting opioids with an average increase of 8.1 units, reflecting ongoing pain or greater disability among participants receiving opioid medications. Time spent in the SSU was associated with the severity of disability. Patients who later developed moderate or severe disability spent a mean of 31 h in SSU, compared to 17.5 h among those with no or mild disability (*p* = 0.012). Only 45% of patients sought ongoing physiotherapy care in the month following discharge.

**Conclusion:**

At one month postdischarge, most participants reported considerable ongoing disability and pain. A substantial proportion continued to use opioids, while physiotherapy services remained underutilised. Findings from this study informed a local business case to establish physiotherapy ‘virtual hot clinics’ for patients within 1 week of discharge.

## 1. Background

Nontraumatic low back pain (LBP) is a prevalent health issue in Australia, with 79.2% of adults experiencing it at some point in their lives [1]. LBP accounts for up to 4% of emergency department (ED) visits in Australia [2–4], creating a substantial economic burden. A recent study found that each episode of ED care costs an average of $2959 [5]. LBP also imposes considerable disability on sufferers, with 70% of patients reporting functional impairment and 59% reporting moderate‐to‐severe pain 1 week after onset. At 3 months, 48.4% continued to experience functional impairment [6].

Unlike the primary care setting, there is a relative lack of outcome data on patients who present to EDs with LBP [7, 8]. A report of LBP patients accounting for over 120,000 visits by the Australian Institute of Health and Welfare does not provide data on postdischarge outcomes, limiting understanding of recovery trajectories following ED care for this condition [9]. A recent systematic review and meta‐analysis to characterise the clinical course of nonspecific LBP following ED presentation reported high initial pain intensity (mean 71/100), with significant improvement within the first week, suggesting a predictable pattern of recovery, with most improvement occurring early [5]. This finding is not consistent with other studies that show ongoing disability following discharge from the ED. Friedman et al. found that approximately 28% still reported moderate or severe pain at 1 week, with nearly half of the patients (*n* = 120) continuing the use of analgesics and experiencing some functional limitation one week postdischarge [10]. In a recent Australian study, 41% of patients were still taking medication for LBP at 4 weeks, with 16%–50% taking opioids at 4 weeks, the lower percentage in opioid naive patients [11]. This study did not measure disability. In a 1‐year follow‐up of 600 consecutive ED patients with acute LBP pain, only 70% achieved complete recovery, leaving 30% without full recovery at 12 months, while 27% had not recovered from pain and 14% had not recovered from disability within one year [12].

The primary aim of the study was to evaluate the recovery of ED patients admitted to the short stay unit (SSU) for the management of LBP by following them up 1 month after discharge. The study objectives were to

- Investigate the recovery path of LBP patients discharged from the SSU.
- Explore postdischarge management and follow‐up practices.
- Assess the level of disability 1 month after SSU attendance.

## 2. Methodology

### 2.1. Study Design and Setting

*Study Design*: This quantitative study used a cross‐sectional observational design.

*Setting*: The study was conducted in a 670‐bed metropolitan quaternary hospital with 90,000 ED visits annually. The ED includes a 20‐bed SSU for patients requiring further care with an anticipated length of stay under 24 h. Most SSU patients are discharged home, returned to residential aged care or transferred to a subacute care facility; approximately 9.5% are admitted to an acute inpatient ward, and a few are transferred to a private facility.

The SSU is staffed by ED nurses and a medical team 24/7, with medical care provided by an ED consultant for 18 h a day, with overnight coverage by a resident. Pharmacy and care coordination services are available from 8:00 a.m. to 9:00 p.m. daily. The unit lacks dedicated physiotherapy, psychological or occupational therapy services.

### 2.2. Study Population

Patients were identified using DRG‐10 coding for LBP, such as M54.5 (LBP), M51.362 and M51.372 (intervertebral disc degeneration) [13]. Exclusion criteria were acute trauma pain, cancer‐related pain and patients under 18 years. No a priori sample size calculation was performed.

Using convenience sampling, a monthly list of SSU admissions for nontraumatic LBP or sciatica between June 2023 and February 2024 was generated by the hospital’s business intelligence unit. A total of 422 participants were identified and invited to participate in the survey. Initially, surveys were mailed within 1 month of discharge; following poor response rates and subsequent ethics approval, participants were contacted by telephone (*n* = 78) or offered a text message with a link to the survey (*n* = 13). Four patients were excluded as their presentations were not due to atraumatic LBP pain, as found before advancing their recruitment, leaving 89 participants (21%) in the final cohort.

### 2.3. Data Sources and Data Collection

Clinical and demographic data were extracted from the hospital’s electronic medical record (EMR). Postdischarge information was self‐reported using a purpose‐designed questionnaire. Functional data were collected using the Modified Oswestry LBP Disability Questionnaire (MOQ), a validated 10‐item tool used to assess the degree of disability and functional impairment in individuals with LBP [14]. Each section is scored on a scale from 0 to 5, with higher scores indicating greater levels of disability, yielding a total Sum Score ranging from 0 to 50, with higher scores indicating greater disability. Scores were categorised into disability levels: no disability (0–4), mild disability (5–14), moderate disability (15–24), severe disability (25–34) and complete disability (35–50) [14]. All data were entered into Microsoft Excel and imported into SPSS for statistical analysis.

### 2.4. Statistical Analysis

Descriptive analyses were used to summarise sociodemographic and clinical characteristics. Missing data in the MOQ were assessed using Little’s test for Missing Completely at Random (MCAR) [15]. The test was not statistically significant (*χ* ^2^ = 44.967, df = 52, *p* = 0.744), indicating the missing data were consistent with an MCAR pattern. Under this assumption, missing values in continuous variables were imputed using mean substitution, and missing values in categorical variables were imputed using the mode when the proportion of missing data was less than 5% [16–18]. Following imputation, 89 participants were retained in the analytical dataset.

Treatment response variables (pharmacological and nonpharmacological) were collapsed from a four‐point Likert scale into two categories: ‘worse or neutral’ and ‘improved or much improved’ to address low cell counts in several categories. Disability was initially classified into five levels: no (0–4), mild (5–14), moderate (15–24), severe (25–34) and complete disability (35–50), but was collapsed into two groups for bivariate comparison to allow adequate cell sizes and maintaining clinical relevance no/mild disability and moderate/severe disability (of which five self‐reported being ‘completely’ disabled one month postdischarge).

An unweighted bivariate analysis was conducted to examine associations between Disability Levels (no, mild, moderate, severe and complete disability) and selected sociodemographic and clinical variables. Associations between categorical variables and disability levels were examined using the Chi‐square test, with Fisher’s exact test applied when expected cell counts were small. For continuous variables (age and time in SSU length of stay), comparisons across disability levels were performed using the Mann–Whitney *U* test.

Multiple linear regression analyses were used to explore predictors of the Oswestry Sum Score. Regression modelling was performed only when sample size, variability and diagnostic checks indicated model stability. Models that produced unstable estimates or were not statistically meaningful were not interpreted further. To detect a moderate effect size (*f* ^2^ = 0.15) with 80% power and a 5% significance level, a minimum of 84 participants will be needed based on a regression model with 5 covariates [19]. Therefore, a sample size of 89 is sufficient to provide adequate statistical power for the study.

Assumptions related to all statistical methods deployed were validated using model fitting statistics, including residual plots, variance inflation factors (VIFs) and Durbin–Watson (DW) statistics. All analyses were conducted using IBM SPSS Version 29 and *R* 4.0.2, with a significance level of 0.05 applied to all tests.

### 2.5. Ethics

The study was approved by the institutional Human Research Ethics Committee (HREC/96718/Austin‐2023). Consent was waived for the observational component of the study, while for the completion of the questionnaire, consent was sought and obtained from the participants after providing them participant information sheet.

## 3. Results

The mean age of the participants was 63.4 years (SD = 19.4), ranging from 20.3 to 96.1 years. The average SSU length of stay was 27.1 h (median = 17.2 h, IQR = 17.7; range 3.9–175.8 h). The mean Oswestry Sum Score was 22.2 (SD = 12.6; range 0–44), reflecting an overall moderate level of disability. More than half of the participants were female (58.0%, *n* = 52). Approximately one‐quarter of participants (25.8%, *n* = 23) were admitted to hospital after their Short Stay Ward admission. Among those admitted, 43.5% (*n* = 10) were admitted on the same day, 34.8% (*n* = 8) within 1‐2 days and 8.7% (*n* = 2) after ≥ 3 days, while for 13.0% (*n* = 3) timing was not reported (Table 1).

**TABLE 1:** Sociodemographic and clinical characteristics of the participants (*N* = 89).

Variable	N/mean (median)	Percentage/SD (IQR) [min–max]
Age (in years)^∗^	63.39	19.36 [20.3–96.1]
Time in SSU LOS (in hours)^∗∗^	17.20 (17.20)	17.70 [3.9–175.8] (17.70)
Sum score^∗^	22.16	12.57 [0–44]
Gender		
Male	37	42.0
Female	52	58.0
Occupation		
Unemployed	2	2.2
Labourer	13	15.0
Retired/home duty	38	43.0
Education teacher	4	4.5
HCW	2	2.2
Desk job	9	10.0
Not responded	21	24.0
Admission to hospital after SSU stay	23	25.8
Same day	10	43.5
Within 1‐2 days	8	34.8
≥ 3 days	2	8.7
Not responded	3	13.0
Follow‐up care sought postdischarge categories		
Physiotherapist	40	45.0
General practitioner	63	71.0
Surgeon (orthopaedic, neuro or other)	13	14.6
Chiropractor	6	6.7
Osteopath	5	5.6
Masseuse	3	3.4
Myotherapist	6	6.7
Radiology services (radiologist/interventional radiologist)	10	11.2
Other healthcare provider	9	10.1
Nonpharmacological treatment of back pain effect		
Heat packs (applied)	44	49.0
Worse	1	2.3
Neutral	12	27.3
Improved	25	56.8
Much improved	6	13.6
Muscle massage (done)	33	37.0
Worse	1	3.0
Neutral	6	18.2
Improved	19	57.6
Much improved	7	21.2
Electrical stimulation/TENS (applied)	15	17.0
Worse	1	6.7
Neutral	7	46.7
Improved	6	40.0
Much improved	1	6.7
Strapping (applied)	11	12.0
Worse	0	0.0
Neutral	5	45.5
Improved	4	36.4
Much improved	1	9.1
Not responded	1	9.1
Spinal injection (applied)	10	11.0
Worse	2	20.0
Neutral	6	60.0
Improved	1	10.0
Much improved	0	0.0
Not responded	1	10.0
Manipulation (done)	9	10.0
Worse	1	11.1
Neutral	0	0.0
Improved	5	55.6
Much improved	3	33.3
Operation/surgery (done)	6	6.7
Worse	1	16.7
Neutral	1	16.7
Improved	1	16.7
Much improved	2	33.3
Not responded	1	16.7
Acupuncture (done)	6	6.7
Worse	0	0.0
Neutral	1	16.7
Improved	4	66.7
Much improved	1	16.7
Back traction (done)	2	2.2
Worse	0	0.0
Neutral	1	50.0
Improved	1	50.0
Much improved	0	0.0
Ultrasound therapy (done)	1	1.1
Worse	0	0.0
Neutral	1	100.0
Improved	0	0.0
Much improved	0	0.0
Other treatment (yes)	14	15.7
Pharmacological treatment of back pain effects		
Paracetamol (used)	62	70.0
Worse	0	0.0
Neutral	22	35.5
Improved	31	50.0
Much improved	8	12.9
Not responded	1	1.6
Nonsteroidal analgesic (used)	28	31.0
Worse	0	0.0
Neutral	9	32.1
Improved	17	60.7
Much improved	1	3.6
Not responded	1	3.6
Short‐acting opioid (used)	29	33.0
Worse	1	3.4
Neutral	6	20.7
Improved	15	51.7
Much improved	7	24.1
Long‐acting opioid (used)	21	24.0
Worse	0	0.0
Neutral	5	23.8
Improved	11	52.4
Much improved	5	23.8
Disability levels		
No disability (0–4)	10	11.2
Mild disability (5–14)	16	18.0
Moderate disability (15–24)	25	28.1
Severe disability (25–34)	21	23.6
Complete disability (35–50)	17	19.1

^∗^Mean and SD for symmetric metric variables.

^∗∗^Median and IQR for nonsymmetric metric variable.

### 3.1. Postdischarge Healthcare Utilisation

**FIGURE 1:**
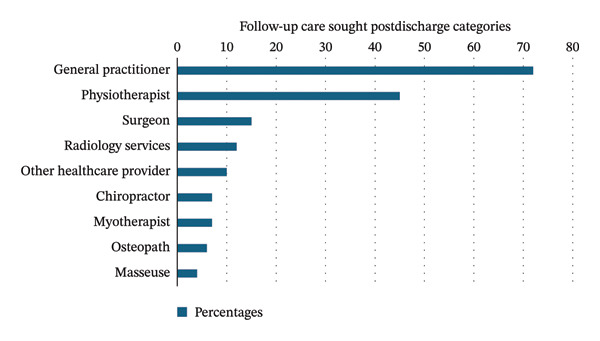
Percentage of participants who reported seeking each type of follow‐up care after discharge from the short stay unit (yes responses only) (*N* = 89).

General Practitioners were the most frequently consulted providers, with 71% (*n* = 63) seeking GP care after discharge. Less than half of the participants consulted a physiotherapist (45.0%, *n* = 40). Use of chiropractors, osteopaths, massage therapists and myotherapists was low, each accounting for < 7% of respondents (Figure 1).

### 3.2. Nonpharmacological Treatments

**FIGURE 2:**
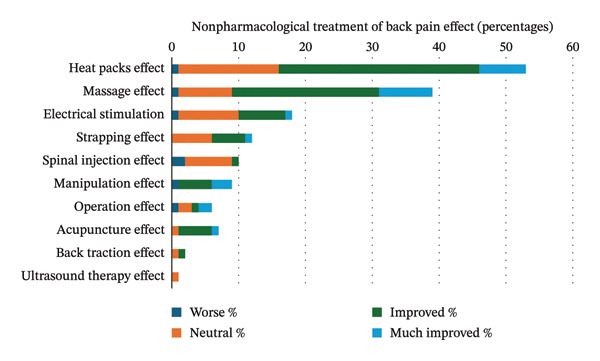
Distribution of perceived effects of nonpharmacological treatments for back pain among participants who received each treatment (*n* = 89).

Heat packs were the most frequently used treatment (49.0% (*n* = 44). Among users, 56.8% (*n* = 25) indicated improvement and 13.6% (*n* = 6) reported much improvement. Muscle massage was used by 37.0% (*n* = 33) of participants, with 57.6% (*n* = 19) reporting improvement and 21.2% (*n* = 7) reporting much improvement. Electrical stimulation was used by 17.0% (*n* = 15), with mixed responses: 40.0% (*n* = 6) reported improvement, 46.7% (*n* = 7) reported a neutral effect and 6.7% (*n* = 1) reported worsening. Other treatments were less commonly used: strapping (12.0%, *n* = 11), spinal injections (11.0%, *n* = 10), manipulation (10.0%, *n* = 9), surgery (6.7%, *n* = 6), acupuncture (6.7%, *n* = 6), back traction (2.2%, *n* = 2) and ultrasound therapy (1.1%, *n* = 1) (effects of nonpharmacological treatments; Figure 2 and Table 2).

**TABLE 2:** Regression coefficient for the association between the sum score and selected predictors (based on pharmacological treatments received).

Predictors	Unstandardised regression coefficient (95% CI)	Standard error	Standardisation regression coefficient	*t*‐value	*p* value	VIF
Constant	14.448 (9.59–19.31)	2.442		5.916	< 0.001	
Paracetamol	5.427 (2.35–13.86)	2.817	0.197	1.926	0.057	1.107
Nonsteroidal analgesics	−1.719 (−7.16–3.72)	2.736	−0.065	−0.628	0.532	1.115
Short‐acting opioid	7.483 (−0.18–11.03)	2.634	0.284	2.842	0.006	1.052
Long‐acting opioid	8.106 (2.25–12.72)	2.894	0.279	2.801	0.006	1.042

*Note:* DW = 1.93, multiple *R* squared = 0.203, *F*‐statistic = 5.34 and *p* < 0.001.

Outcome based on percentage calculated using only those who received the respective treatment as the denominator. After collapsing the four treatment effect categories into two groups (worse or neutral vs improved or much improved), the majority of participants reported improvement across most nonpharmacological treatments, except for ultrasound therapy and spinal injection.

### 3.3. Pharmacological Treatment

**FIGURE 3:**
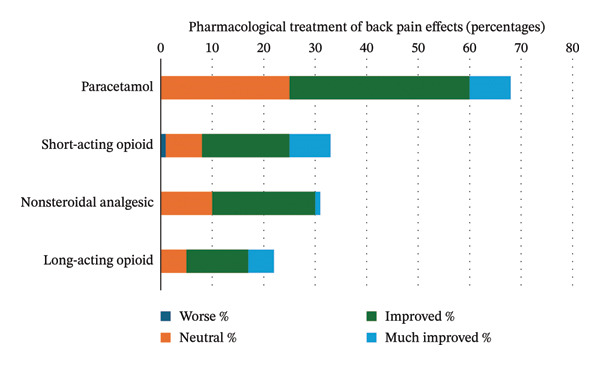
Distribution of perceived effects of pharmacological treatments for back pain among participants (*N* = 89).

Paracetamol was the most frequently used medication (70.0% *n* = 62), with half (*n* = 31) reporting improvement and 12.9% (*n* = 8) much improvement. Nonsteroidal analgesics (NSAIDs) were used by 31.0% (*n* = 28), with 60.7% (*n* = 17) reporting improvement. Short‐acting opioids were used by 33.0% (*n* = 29), and long‐acting opioids by 24.0% (*n* = 21), with 50.6% (*n* = 45) using either form. Across opioid users, more than half reported improvement or much improvement (Figure 3).

Outcome based on the percentage of patients reporting each category (worse, neutral, improved and much improved). Percentage calculated using only those who received the respective treatment as the denominator. After collapsing the four treatment effect categories into two groups (worse or neutral vs improved or much improved), most participants reported improvement across all pharmacological treatments.

### 3.4. Time Spent in SSU

Time spent in the SSU was associated with the severity of disability. Patients who later developed moderate or severe disability spent a mean of 31 h in SSU, compared with 17.5 h among those with no or mild disability (*p* = 0.012).

### 3.5. Disability Levels

Most participants self‐reported experiencing moderate‐to‐severe disability. Specifically, 28.1% (*n* = 25) were classified as having moderate disability, 23.6% (*n* = 21) as severe disability and 19.1% (*n* = 17) as complete disability. Smaller proportions were classified as having mild disability (18.0%, *n* = 16) or no disability (11.2%, *n* = 10). A detailed breakdown of the participants’ sociodemographic and clinical characteristics is provided in Table 1.

The unweighted bivariate analyses (Table 3) showed that most sociodemographic characteristics, follow‐up care variables, nonpharmacological treatment effects and pharmacological treatment effects were not significantly associated with disability levels. This included physiotherapy, GP visits, surgeon review, chiropractic, osteopathy, myotherapy, radiology services and all nonpharmacological treatment effect categories (all *p* > 0.05). Similarly, the effects of paracetamol, nonsteroidal analgesics and long‐acting opioids were not significantly associated with disability level. Gender was significantly associated with disability levels (*χ* ^2^ (1) = 3.93, *p* = 0.047), with a higher proportion of females (78.8%) classified in the moderate‐to‐complete disability group compared with males (59.5%). In addition, the effect of short‐acting opioid use showed a statistically significant association with disability levels (Fisher’s exact test, *p* = 0.010). Among participants who reported an improved or much improved response to short‐acting opioids, all were classified in the moderate‐to‐complete disability group, whereas participants reporting worse or neutral effects were more evenly distributed across disability categories. No significant differences in mean age or length of stay in the SSU were observed across disability categories (*p* > 0.05), indicating that these continuous variables were not associated with disability levels.

**TABLE 3:** Bivariate (unweighted) associations of disability levels with the selected sociodemographic and clinical characteristics (*N* = 89).

Variable	Disability levels		*p* value (Cramer’s V/r)
	No or mild disability *N* (%)	Moderate‐to‐complete disability *N* (%)	
Gender (*N* = 89)			
Male	15 (40.5)	22 (59.5)	0.047 (0.210)
Female	11 (21.2)	41 (78.8)	

Physiotherapist (*N* = 89)			
Yes	11 (27.5)	29 (72.5)	0.748 (0.034)
No	15 (30.6)	34 (69.4)	

General practitioner (*N* = 89)			
Yes	15 (23.8)	48 (76.2)	0.081 (0.185)
No	11 (42.3)	15 (57.7)	

Surgeon (*N* = 89)			
Yes	1 (7.7)	12 (92.3)	0.098^∗∗^ (0.196)
No	25 (32.9)	51 (67.1)	

Chiropractor (*N* = 89)			
Yes	2 (33.3)	4 (66.7)	1.000^∗∗^ (0.024)
No	24 (28.9)	59 (71.1)	

Osteopath (*N* = 89)			
Yes	3 (60)	2 (40)	0.147^∗∗^ (0.165)
No	23 (27.4)	61 (72.6)	

Myotherapist (*N* = 89)			
Yes	2 (33.3)	4 (66.7)	1.000^∗∗^ (0.538)
No	24 (28.9)	59 (71.1)	

Radiology services (*N* = 89)			
Yes	3 (30.0)	7 (70.0)	1.000^∗∗^ (0.006)
No	23 (29.1)	56 (70.9)	

Heat packs effect (*n* = 44)			
Worse or neutral	2 (15.4)	11 (84.6)	0.170^∗∗^ (0.228)
Improved or much improved	12 (38.7)	19 (61.3)	

Massage effect (*n* = 33)			
Worse or neutral	1 (14.3)	6 (85.7)	0.195^∗∗^ (0.295)
Improved or much improved	13 (50.0)	13 (50.0)	

Electrical stimulation effect (*n* = 15)			
Worse or neutral	1 (12.5)	7 (87.5)	0.119^∗∗^ (0.472)
Improved or much improved	4 (57.1)	3 (42.9)	

Strapping effect (*n* = 11)			
Worse or neutral	1 (16.7)	5 (83.3)	0.545^∗∗^ (0.261)
Improved or much improved	2 (40.0)	3 (60.0)	

Paracetamol effect (*n* = 64)			
Worse or neutral	5 (22.7)	17 (77.3)	0.752^∗∗^ (0.043)
Improved or much improved	8 (19.0)	34 (81.0)	

Nonsteroidal analgesic effect (*n* = 28)			
Worse or neutral	2 (22.2)	7 (77.8)	1.000^∗∗^ (0.013)
Improved or much improved	4 (21.1)	15 (78.9)	

Short‐acting opioid effect (*n* = 29)			
Worse or neutral	3 (42.9)	4 (57.1)	0.010^∗∗^ (0.602)
Improved or much improved	0 (0.0)	22 (100.0)	

Long‐acting opioid effect (*n* = 21)			
Worse or neutral	1 (20.0)	4 (80.0)	0.429^∗∗^ (0.200)
Improved or much improved	1 (6.3)	15 (93.8)	

	*Median (IQR)*	*Median (IQR)*	

Age (in years)^∗^	65.95 (31.25)	70.20 (37.50)	0.321 (0.110)

Time in SSU LOS (in hours)^∗^	16.35 (16.75)	20.50 (17.30)	0.248 (0.120)

^∗^Metric variables analysed using Mann–Whitney *U* test.

^∗∗^ *p* values derived from Fisher’s exact test when chi‐square assumptions were violated.

Several exploratory regression models were initially examined to evaluate the association between different groups of clinical predictors and the sum score. However, only the model based on pharmacological treatments received demonstrated a statistically significant overall fit and stable parameter estimates. The other models, including those based on follow‐up care pathways, nonpharmacological treatments and treatment effects, were not statistically significant.

The overall regression model examining the association between pharmacological treatments and the sum score was statistically significant (*F* (4, 84) = 5.34, *p* < 0.001) (see Table 2). The multiple R‐squared value of 0.203 suggests that the combined independent variables explained only 20.3% of the variance in the sum score. The overall explanatory power of the regression model is modest (R‐square = 20.3%), which is common in social science, health and human behavioural studies [20]. These variables included paracetamol, nonsteroidal analgesics, short‐acting opioids and long‐acting opioids. Receipt of short‐acting opioids was significantly associated with higher disability scores. This means that participants who received short‐acting opioids had, on average, sum scores about 7 units higher than those who did not receive these medications, indicating greater disability. Similarly, receipt of long‐acting opioids was also significantly associated with higher sum scores, suggesting an average increase of about 8 units in disability scores among those who received long‐acting opioids. Paracetamol use showed a positive association with the sum score, indicating an average increase of about 5 units in disability scores. However, this result was only marginally significant (*p* = 0.057) and should be interpreted with caution. No significant association was observed for nonsteroidal analgesics.

Before finalising the model, standard diagnostic checks were performed. The normality of residuals was assessed using normal probability (P–P) plots of standardised residuals, which showed that most points lay close to the reference line, supporting the assumption of normality. Multicollinearity was evaluated using variance inflation factors (VIF), and no evidence of problematic multicollinearity was observed (Table 2). The DW test was used to assess autocorrelation, with values between 1.5 and 2.5 considered acceptable; the observed DW value of 1.93 indicated independence of residuals and no evidence of serial correlation. Homoscedasticity was examined using residuals versus fitted values plots, and the random dispersion of residuals supported the assumption of constant variance.

## 4. Discussion

The primary aim of the study was to evaluate the status of ED patients admitted to SSU for the management of LBP 1 month after discharge. In this study, most patients still had moderate to complete disability, as assessed by the validated Modified Oswestry LBP Disability tool [14, 21], with considerable impact on personal care and lifestyle. This includes limitations in lifting, walking distance, sitting or standing for more than half an hour, and sleeping. Social life is commonly restricted, as is travel; less than half of our participants had returned to full work or home duties.

Most patients sought follow‐up with a general practitioner, while fewer than half (44.9%) saw a physiotherapist, although an additional 22% attended alternative practitioners. The benefit of services like physiotherapy in managing LBP cannot be underestimated [22], raising the need to re‐evaluate our patient education and referral process. Our study investigated which nonpharmacological modalities patients found useful postdischarge. Simple heat packs were commonly used, with the large majority reporting benefit. However, because of safety concerns, heat packs were removed from our department a while ago. Muscle massage was sought by over 33% of patients, with most reporting benefit. The few who had spinal manipulation mostly reported benefit. Most doctors and nurses are not trained in muscle massage and likely feel it is outside their scope of practice, although the technique is not complex, if time‐consuming. In contrast, spinal manipulation requires specialist training. Thus, there are considerable barriers to using nonpharmacological therapies, ranging from fear of burns, scope of practice, time constraints and lack of additional funding for SSU physiotherapy. This has forced reliance on bed rest and analgesia as treatment modalities. Our study questionnaire did not specifically enquire about exercise.

The Australian Commission on Safety and Quality in Healthcare recently published the LBP Clinical Care Standard (LBPCCS) [11, 23], which highlights the importance of a comprehensive initial clinical and psychosocial assessment, patient education, allied health referral, encouragement of self‐management, maintenance of physical activity and appropriate physical, psychological and nonpharmacological interventions. The standard also emphasises judicious use of analgesia and the avoidance of opioids. A recent study examining adherence to these guidelines 12 months after their release reported that compliance remained highly variable. In the ED setting, patients were still more likely to receive imaging and pharmacological treatments (including opioids) than the recommended nonpharmacological interventions [8, 24]. Post hoc comparison with the LBPCCS suggests that our service does not meet several key requirements, particularly regarding opioid avoidance, given the high‐risk and low‐benefit ratio [25]. Notably, 57% of patients in our cohort were still taking a form of opioid at follow‐up, often in combination with other medications. This may reflect greater symptom severity, supported by the significant association with disability observed in this group. Nevertheless, the proportion of patients using opioids remains higher than that reported in the literature [26]. Nevertheless, 75% of patients reported improvement or much improvement with short‐ or long‐acting opioids. The low number of patients reviewed by a physiotherapist is a concerning finding.

The results of our study have informed a business case to enhance postdischarge follow‐up. Electronic referral to our specialist clinics will now occur, enabling patients to be contacted by a physiotherapist through a Virtual Hot Clinic within a week of discharge. This process is intended to ensure that the key elements of the LBPCCS are initiated in a timely and consistent manner.

### 4.1. Study Limitations

The initial email yielded a low response rate, but this improved with follow‐up phone calls and interviews. The most common issue in contacting patients was the call being redirected to voicemail. It is possible that the patients who completed the survey were those unable to return to work, thus representing the more severe cases of back pain.

Our study relies on self‐reporting. While this may introduce some degree of reporter bias, we view it as a strength. Understanding our patients’ perspectives is essential to achieving our goal of gaining insight into the recovery process. The use of a validated disability scale reinforces the reliability of our findings. Recall bias was minimised by having the survey completed within a mean time of 36 days of discharge.

We did not collect data on prior opioid usage or whether opioids were first administered in the ED before discharge. While this is of interest, it would not impact our primary objective of understanding the degree of self‐reported disability. Due to its observational design, causal inferences cannot be drawn.

As a single‐site study with clinical management not adhering to the standard, our findings may not be reproducible or generalisable in other settings. However, our results seem to be consistent with other studies [23, 27].

This study did not specifically evaluate the economic impact of LBP. However, given the degree of disability at the time of survey completion, our study is consistent with others that demonstrate LBP comes with a considerable socioeconomic burden [28].

Given the modest explanatory power of the regression model due to low explained variance (R‐square), the findings should be interpreted cautiously to avoid overstating their practical significance. This may be due to the absence of potentially important confounders (e.g., comorbidities and baseline disability) from the model, as these variables were not available in the dataset, potentially leading to residual confounding.

A key limitation of this cross‐sectional observational study is that exposure and outcome were measured simultaneously, preventing the establishment of a temporal sequence and limiting the ability to infer causal relationships.

## 5. Conclusion

Patients discharged from our SSU with LBP continue to experience significant disability, affecting both work and lifestyle. There is a higher‐than‐expected reliance on opioids, and fewer than half of the patients were followed up with a physiotherapist in the community.

Our service does not meet the best practice guidelines outlined in the Clinical Care Standard for LBP management (ACSQHC). The findings from this research have supported a business case to improve follow‐up of patients with LBP discharged from our SSU. We recommend future studies that compare responders to nonresponders for validation on whether patients with worse outcomes are more motivated to respond.

## Funding

No funding was received for this manuscript.

Open access publishing facilitated by Victoria University, as part of the Wiley ‐ Victoria University agreement via the Council of Australasian University Librarians.

## Conflicts of Interest

The authors declare no conflicts of interest.

## Data Availability

The data that support the findings of this study are available from the corresponding author upon reasonable request.
